# Prevalence of Insulin Resistance in Urban Indian School Children Who Are Overweight/Obese: A Cross-Sectional Study

**DOI:** 10.3389/fmed.2021.613594

**Published:** 2021-02-12

**Authors:** Rashmi Ranjan Das, Manaswini Mangaraj, Saurav Nayak, Amit Kumar Satapathy, Samarendra Mahapatro, Jagdish Prasad Goyal

**Affiliations:** ^1^Department of Pediatrics, All India Institute of Medical Sciences (AIIMS), Bhubaneswar, India; ^2^Department of Biochemistry, All India Institute of Medical Sciences (AIIMS), Bhubaneswar, India; ^3^Department of Pediatrics, All India Institute of Medical Sciences (AIIMS), Jodhpur, India

**Keywords:** insulin, pediatric, metabolic syndrome, observational study (cross-sectional study), HOMA-IR

## Abstract

**Background:** Limited data are available for insulin resistance (IR) in over-weight/obese children from the Indian subcontinent. Identifying predictors of IR in this population is important, as they may be used as a screening tool for future metabolic complications.

**Materials and Methods:** This school-based cross-sectional study was conducted in an Eastern Indian city. Anthropometry and blood pressure measurements were carried out as per the published guidelines. Venous blood samples were taken in a fasting state to measure plasma glucose, insulin, and lipid profile. IR was measured quantitatively by a homeostatic model of assessment (HOMA-IR).

**Results:** A total of 545 (28.2%) children who were overweight or obese were included. The male:female ratio was 1:1.27. The overall prevalence of metabolic syndrome (MS) in these children was 21.8%. Around 32.3% of children had HOMA-IR of ≥2.5, and 22.2% had HOMA-IR of ≥3.16. The mean HOMA-IR in children with MS was 5.46 compared to 2.18 in those without MS. An increased risk of IR with low HDL, high triglyceride, increased waist circumference, and increased BP (both systolic and diastolic) was found. This means that insulin resistance was more common in children who were overweight or obesity and had underlying MS.

**Conclusions:** The present school-based study found a high prevalence of insulin resistance among children who were overweight or obese. This could predict an increased risk of future adverse cardio-vascular events in the studied children. The findings of this study would help in planning and implementing primary prevention programs targeting weight management and lifestyle change in schoolchildren.

## Introduction

There is a steady increase in the prevalence of being overweight and having obesity among children and adolescents because of faulty dietary habits and lack of exercise (1). The World Health Organization (WHO) in year 2016 estimated the prevalence to be 41 million in children <5 years of age and >340 million in children and adolescents >5 years of age (1). The prevalence is increasing in developing countries, like the Asian sub-continent, due to dramatic changes in lifestyle and economic growth (1). In a populous country like India, the prevalence of overweight or obese citizens has nearly doubled in the last two decades (2, 3).

Childhood obesity is associated with an increased risk of insulin resistance, type 2 diabetes mellitus (DM), metabolic syndrome (MS), and cardiovascular diseases (CVD) in young adulthood (4). Insulin resistance (IR) is a common phenomenon in South Asian populations and is associated with characteristic phenotypic features (upper body adiposity, low muscle mass, and increased body fat) (5). In a previously published study from India, the authors noted a higher prevalence of insulin resistance in post-pubertal urban Asian adolescents (14–25 years of age) with overall obesity as well as abdominal and truncal obesity (6). Insulin resistance may be the physiologic disturbance underlying the subsequent development of metabolic syndrome (MS) characterized by hyperglycemia, dyslipidemia, and hypertension (5). A recent study including schoolchildren of 6–16 years of age from India found a higher prevalence of MS (21.8%) (3).

In an obesity setting, insulin resistance (IR) has a shared pathophysiology of dysmetabolic status with several conditions and is believed to be the main mechanistic underpinning of obesity (7). Decreased tissue response to insulin-mediated cellular actions and lipolytic action in adipose tissue with free fatty acid (FFA) accumulation in the liver contributes to IR development. The inflammatory cytokines from dysfunctional adipocytes also mediates the inflammation associated with IR (8). This IR is more prevalent in obese children, being related to Beta-cell function resulting from ectopic fat deposition (9). It is more common in adolescents than adults with a similar degree of adiposity and glycemic status, moving toward a faster rate of decline in Beta-cell function. Puberty, a physiological state in this age group, contributes to IR in children (10). As there is no clear-cut idea to define IR in children, and Fasting Plasma Insulin is a poor measure of Insulin sensitivity, IR is an important issue in children confronting clinicians (11).

Limited data are available for IR and its association with MS in children from the Indian subcontinent. Identifying predictors of IR in children who are overweight or obese is important, as they may be used as a screening tool for future metabolic complications like Type 2 DM or CVD (5). Therefore, the present study was designed to measure the prevalence of IR in overweight/obese schoolchildren.

## Materials and Methods

This cross-sectional study included schoolchildren of aged 6–16 from Bhubaneswar, an Eastern Indian city, from April 2017 to March 2018. Children with serious underlying diseases, diabetes mellitus, or who were taking drugs that could cause overweight/obesity/metabolic syndrome were excluded.

Children were enrolled in the study after getting permission from the dean/principal, and after obtaining consent from the parents. Clinical and demographic details were recorded in case record forms, and physical examination was done to look for any abnormality. Weight, height, and waist and hip circumference were measured by trained research staff. Weight measurement was taken in a tarred weight machine (to nearest 100 g), and height measurement was taken by a stadiometer [to nearest centimeter (cm)]. The hip circumference (cm) measurement was taken at the prominence of the buttocks. The waist circumference (cm) measurement was taken while the child was standing, with the help of a non-stretchable (plastic) measuring tape in the mid-axillary line at the end of expiration, at midpoint between the costal margin and iliac crest. Hip and Waist circumference cut-offs used the data of Indian children (12). A standardized sphygmomanometer with appropriately sized cuffs was used for blood pressure (BP) measurement (an average of readings was taken). The American Academy of Pediatrics guideline was used to define hypertension (13). Body mass index (BMI) was calculated (in kg/m^2^) and interpreted as per the method described for Indian children (14). As per the revised IAP (Indian Academy of Pediatrics) classification, overweight and obesity were defined as an adult equivalent of 23 and 27 cut-offs, respectively.

Physical examination was done for the presence of acanthosis nigricans (on the neck, axillae, and skin folds). Liver span was measured by the distance between upper border (percussion) and lower border (palpation method) to note any hepatomegaly. Fasting venous blood samples were collected from children classified as overweight or obese for the measurement of following parameters: blood glucose, lipid profile (triglyceride, HDL, and LDL cholesterol), and insulin. Any deviation in the measured value was defined as per the Indian data (15, 16). Plasma glucose was measured by Glucose oxidase technique, and a Beckman Coulter (AU5800 Clinical Chemistry Analyzer, Danaher Corporation, California, USA) analyzed the lipid profile. Fasting insulin level (FPI) was measured by a Beckman Coulter (Access 2 Immunoassay System, Danaher Corporation, California, USA).

We followed The International Diabetic Federation (IDF) criteria to define MS (17). The IDF criteria has not defined MS in children aged 6–10, though it defines central obesity (WC ≥90th centile) in this age group. Various publications have suggested that MS definition by IDF can be extrapolated to children of 6–10 years of age for calculation of MS prevalence, though limitations are there for defining cut-offs for other MS components (18, 19). We extended the IDF criteria to this age group in our study population. We applied IDF obesity criteria in overweight children (BMI ≥85th centile) if central obesity (WC ≥90th centile) was present. We determined insulin resistance (IR) by HOMA-IR, a homeostatic model assessment (12). HOMA-IR is calculated by the following formula: fasting plasma insulin (mIU/L) multiplied by fasting plasma glucose (mmol/L), divided by 22.5 (20). We defined IR as a HOMA-IR score of ≥2.5 as defined previously in urban Indian adolescents (21). We also used another HOMA-IR score of ≥3.16 to define IR, and hyperinsulinemia as a fasting insulin level of > 17 mIU/L as described from studies outside India to note the difference in the prevalence for these cut-offs in Indian children (22, 23). The study was approved by the Institute Ethics Committee of AIIMS Bhubaneswar.

### Statistical Analysis

We used cluster random sampling method (cluster being schools) for sample size calculation. A previous study from Bhubaneswar estimated the mean prevalence of overweight/ obesity in schoolchildren to be of 28% (24). Considering the prevalence of MS to be 20% in overweight/ obese children, the prevalence of MS in normal weight children would be 5.6% (25). To arrive at this, a final sample size was calculated as 2,235 (keeping an absolute error of 2%, multiplying by 4 because of cluster design method, and accounting for an attrition rate of 10%).

Data were entered into a Microsoft Excel spreadsheet, and analyzed using STATA 16.0 software. Means were compared using independent t-test, and proportions were compared using chi-square test. Median values of various parameters were compared between the groups with a presence or absence of IR. Univariate analysis was done using variables that could influence HOMA-IR. A p <0.05 was considered as statistically significant. Correlation between various parameters (anthropometric and metabolic) and HOMA-IR score, as well as between FPI and IR, was calculated using Spearman's rank correlation coefficient (ρ).

## Results

**Figure 1:**
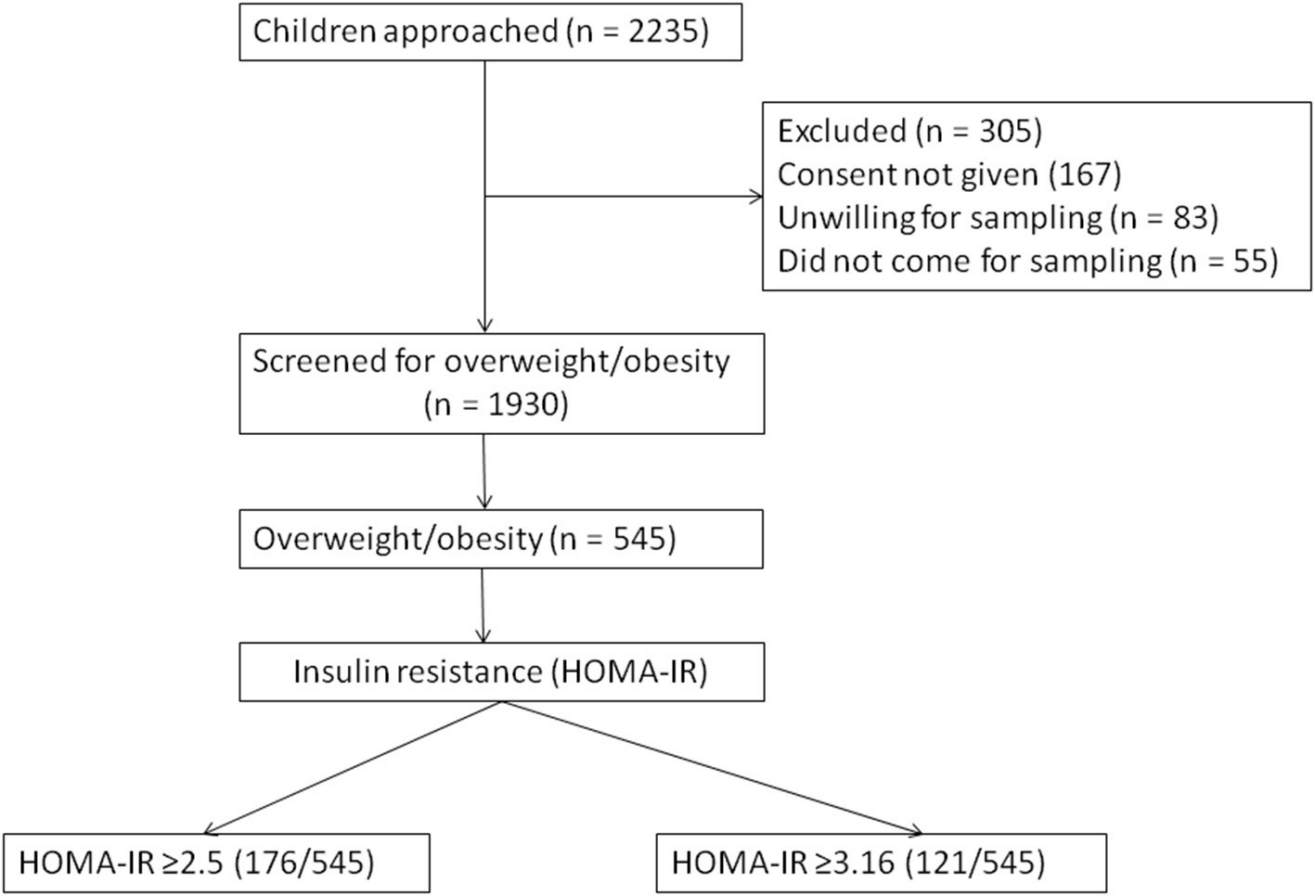
Flow of study participants.

A total of 1,930 children were included and 305 (13.6%) were excluded (due to either not giving consent for blood sampling or for unwillingness to participate) from the study (Figure 1). About 545 children were found to be overweight or obese (overweight = 383, obese = 162). The age distribution was as follows: <10 years age (*n* = 244) and > 10 years age (*n* = 301). The children from private schools (*n* = 1,328) outnumbered those from government schools (*n* = 602). The overall prevalence of metabolic syndrome (MS) in children who were overweight or obese in the study population was 21.8% (119/545). The physical marker of insulin resistance (acanthosis nigricans) was present in 46.4% of children who were overweight or obese. A family history of diabetes and cardio-vascular diseases was present in 42.7%. Around 68.1% children were exclusively breast fed for 6 months. Hepatomegaly was present in 8.1%.

The serum insulin level (Mean ± SD) was significantly different between those with (22.06 ± 6.95) and without MS (10.47 ± 4.34). Similarly, HOMA-IR was also significantly different between those with (5.46 ± 1.84) and without (2.18 ± 1.0) MS. Around 32.3% of children who were overweight or obese (176/545) had HOMA-IR of ≥2.5, and 22.2% (121/545) had HOMA-IR of ≥3.16. In those with MS, 93% had HOMA-IR of ≥2.5 compared to 18% without MS. All the anthropometric and biochemical parameters were significantly increased/elevated (except HDL cholesterol, exclusive breast feeding for 6 months, and age <10 years) in children with insulin resistance (*P* < 0.001) (Table 1). In the sub-group analysis, HOMA-IR values (mean ± SD) were found to increase with an increase in the body weight in both sexes as follows: boys (normal weight 1.81 ± 1.37 vs. overweight 2.84 ± 1.46 vs. obese 4.57 ± 2.28) and girls (normal weight 1.53 ± 1.15 vs. overweight 2.91 ± 1.92 vs. obese 4.46 ± 2.39). The risk of insulin resistance (IR) with various anthropometric and metabolic parameters in the study population was studied. The variables that were found to be associated with an increased risk of IR were: obesity [RR 1.38 (95% CI 1.14–1.66)], high TG [RR 3.12 (95% CI 2.68–3.62)], increased WC [RR 2.01 (95% CI 1.79– 2.43)], high BP [RR 1.37 (95% CI 1.13–1.66)], and family history of cardio-vascular diseases [RR 1.61 (95% CI 1.33–1.94)]. However, age <10 years, exclusive breast feeding for 6 months, and normal HDL level was associated with a decreased risk of IR (Table 2). Except HDL level, all other metabolic and anthropometric parameters had a significant positive correlation with HOMA-IR (Table 3).

**Table 1:** Anthropometric and metabolic parameters in groups with and without insulin resistance in the study population.

**Parameters**		**IR present** **Median (IQR)** **[HOMA-IR> =2.5]** **[*n* = 242]**	**IR absent** **Median (IQR)** **[HOMA-IR <2.5]** **[*n* = 303]**	**Sig. (2-tailed) [Mann-Whitney *U* Test]**
Age (in years)		11 (9.5–13.5)	8 (6–10)	<0.001
Anthropometric parameters	**Male sex**			
	Weight *Z*-score	1.41 (1.22–2.4)	0.91 (0.74–1.38)	<0.01
	Height *Z*-score	0.97 (0.66–1.01)	0.89 (0.68–1.03)	0.07^*^
	BMI *Z*-score	1.43 (1.29–2.3)	0.88 (0.79–1.56)	<0.01
	WC *Z-*score	1.36 (1.02–2.01)	0.89 (0.63–1.15)	<0.001
	**Female sex**			
	Weight *Z*-score	1.34 (1.19–2.1)	0.86 (0.74–1.58)	<0.01
	Height *Z*-score	0.78 (0.62–1.01)	0.75 (0.61–1.03)	0.09^*^
	BMI *Z*-score	1.41 (1.24–2.1)	0.89 (0.77–1.55)	<0.01
	WC *Z*-score	1.36 (1.02–2.01)	0.89 (0.63–1.15)	<0.01
Hypertension^**^	Present	69 (28.5%)	54 (17.8%)	0.003
	Absent	173 (71.5%)	249 (82.2%)	
Metabolic profile	FBS (mg/dL)	92 (77.5–106.5)	61 (56.5–74.5)	<0.01
	TG (mg/dL)	145 (96–194)	82 (77.5–86.5)	<0.01
	HDL (mg/dL)	40 (32–48)	100 (81–119)	<0.01
	LDL (mg/dL)	89 (70–108)	44 (42.5–45.5)	<0.01
	FPI (mIU/mL)	16.95 (9.78–24.12)	7.7 (7.0–8.4)	<0.01

* P >0.05.

** Expressed in proportions and analyzed by Chi-square test.

*BMI, Body mass index; WC, Waist circumference; HC, Head circumference; BP, Blood pressure; SBP, Systolic BP; DBP, Diastolic BP; FPG, Fasting plasma glucose (normal range: 70–110 mg/dl; coefficient of variation [CV]: 15%), TG, Triglyceride (normal range: 27–185 mg/dl; CV: 19%), HDL, High-density lipoprotein (normal range: 12–86 mg/dl; CV: 19%), LDL, Low-density lipoprotein (normal range: 32–202 mg/dl; CV: 19%), FPI, Fasting plasma insulin (normal range: 3–8 mIU/mL; CV: 21%)*.

**Table 2:** Univariate analysis of IR with anthropometric & metabolic parameters.

**Parameters**	**RR**	**95% CI**	***P***
Age (<10 vs. ≥10)	0.49	0.39–0.61	<0.001
Sex (Male vs. Female)	0.98	0.81–1.18	0.8^*^
BMI (Obese vs. Overweight)^**^	1.38	1.14–1.66	<0.001
Hypertension (Presence vs. absence)	1.37	1.13 – 1.66	0.003
Waist Circumference (>90th centile)^***^	2.01	1.79–2.43	0.012
EBF till 6 months (Yes vs. No)	0.68	0.56–0.81	<0.001
Family History of cardio-vascular diseases	1.61	1.33–1.94	<0.001
Fasting Glucose (>100 mg/dL)	2.63	2.33–2.96	<0.001
TG (≥150 mg/dL)	3.12	2.68–3.62	<0.001
HDL (≥40 mg/dL)	0.33	0.28–0.39	<0.001
Insulin (>17 mIU/L)	3.5	3.01–4.07	<0.001

* P ≥0.05; IR, Insulin resistance; EBF, Exclusive breast feeding.

** Overweight and obesity were defined as per revised IAP 2015 classification (14).

*** *Waist circumference was taken from data published in Indian children (12)*.

**Table 3:** Correlation between various anthropometric and metabolic parameters and HOMA-IR score in the study population by Spearman's Rho (ρ).

**Parameters**	**Spearman's Rho (ρ)**	**Sig. (2-tailed)**
Age (Years)	0.42	0.039
Weight (*Z*-score > +2)	0.59	<0.01
Height (*Z*-score > +2)	0.52	0.012
WC (*Z*-score > +2)	0.64	<0.01
HC (*Z*-score > +2)	0.46	0.028
BMI (*Z*-score > +2)	0.57	<0.01
SBP (>95th centile)	0.42	0.039
DBP (>95th centile)	0.48	0.022
FBS (mg/dL)	0.69	<0.01
TG (mg/dL)	0.68	<0.01
HDL (mg/dL)	−0.56	<0.01
LDL (mg/dL)	0.58	<0.01
Insulin (mIU/mL)	0.98	<0.01

## Discussion

The present school-based cross-sectional study from a city in Eastern India found a higher prevalence of insulin resistance in children who were overweight or obese. Around 32.3% of children who were overweight or obese had HOMA-IR of ≥2.5 (Indian cut-off data), and 22.2% had HOMA-IR of ≥3.16 (Western cut-off data). The present study is unique in that it is the first Indian study to evaluate the prevalence of insulin resistance in a cohort of urban schoolchildren of 6–16 years of age who are overweight or obese. There is a concern regarding the increased prevalence of childhood obesity in Indian children and adolescents as this may indirectly influence the rising prevalence of type 2 DM and cardio-vascular diseases (CVD) not only in adults but also in young adults (21). Insulin resistance (IR) is a common feature of obesity, and the metabolic abnormalities associated with IR can compound the complications of obesity, including the development of metabolic syndrome (MS), type 2 DM, and CVD. Different tools are available to measure both insulin sensitivity and resistance (20, 21). HOMA-IR validation for measuring insulin resistance in children and adolescents has already been done previously (26, 27). In a previously published study from India, HOMA-IR values (mean ± SD) increased proportionately with an increase in the body weight in both sexes as follows: boys (normal weight 1.70 ± 1.44 vs. overweight 2.67 ± 1.41 vs. obese 4.39 ± 2.14) and girls (normal weight 1.21 ± 1.10 vs. overweight 3.19 ± 2.02 vs. obese 4.19 ± 2.52) (21). This was similar to the findings in the present study, even though children of a younger age (<10 years) were included. In a study from Sri Lanka, the mean HOMA-IR was 1.1 and 0.94 for girls and boys, respectively; in obese children it was 2.26 (28). This lower level could be explained by a lower prevalence of overweight (11.3%) and obesity (4.2%) in the study population in contrast to the present study (28.2% overall) (28). This is supported by a study from Australia, which found the prevalence of IR in normal weight and obese children to be of 7.1 and 68.4%, respectively (29). Another finding in the present study was that children in the IR group included more adolescents or pubertal age groups as compared to those in the no IR group. As puberty is a state of insulin resistance, it is not surprising that metabolic parameters were different between the two groups.

In a systematic review on the epidemiology of insulin resistance in children (30), the lowest prevalence of IR (3.1%) was reported by a study from Greece in children of 10–12 years of age (31), and the highest (44%) was in New Zealand in children of 15–18 years of age (32). In the present study, the prevalence was high with two different cut-offs, as mentioned above, and is consistent with the fact that Asian populations have a higher prevalence of insulin resistance. Of the 13 studies included in the systematic review, seven showed the IR prevalence to be higher in girls, three in boys, and one did not find any gender specific difference (30). In the present study, we also did not find any gender specific difference in the prevalence of IR, as supported by other studies from India (4, 21).

We found an increased risk of IR with low HDL, high TG, increased WC, and increased BP (both systolic and diastolic). These alterations represent a pro-atherosclerotic profile in the study children. This was similar to findings in previous studies published within (21) as well as from outside India (9, 33). Though we found an inverse correlation between HDL and IR, another study from India found no correlation between the two (21). One study compared risk factors for IR in children and adolescents (9). This study found obesity as the common risk factor with preterm birth and Tanner stage as additional risk factors in children, and WC as an additional risk factor in adolescents.

In a previous study, increased fasting plasma insulin (FPI) in the light of normal fasting plasma glucose (FPG) indicated that blood glucose levels are not suitable for predicting CVD early (28). IR always precedes abnormal glucose homeostasis in overweight or obese children and adolescents; as a result FPI or HOMA-IR has been suggested as a screening tool for early detection of IR (with or without measurement of fasting serum lipids) (34). Another study has suggested screening children with obesity with FPI and FPG (35). Whether an early intervention in children who are overweight or obese and have underlying IR but no metabolic abnormality can prevent the development of future MS needs to be studied and validated in prospective studies. However, the recent Endocrine Society Clinical Practice Guideline does not recommend measuring insulin values in children who were overweight or obese in predicting the future cardio-vascular risk (36).

Though the present study is one of the largest of its kind conducted in Indian children, including both young children and adolescents, there are some limitations. First, oral glucose tolerance test (OGTT) was not performed to define dysglycemia or diabetes mellitus. Second, data on pubertal changes, physical activity, and dietary pattern were not collected.

## Conclusions

The present school-based study found a high prevalence of insulin resistance (32.3%) among children who were overweight or obese. This could predict an increased risk of future adverse cardio-vascular events in the study children. The findings of this study would help in planning and implementing primary prevention programs targeting weight management and lifestyle change in schoolchildren.

## Data Availability Statement

The original contributions presented in the study are included in the article/supplementary material, further inquiries can be directed to the corresponding author/s.

## Ethics Statement

The studies involving human participants were reviewed and approved by Institute Ethics Committee of AIIMS Bhubaneswar. Written informed consent to participate in this study was provided by the participants' legal guardian/next of kin.

## Author Contributions

RD and JG conceived and designed the experiment. MM and SN performed the experiment and analyzed the data. AS and SM provided comments and technical advice. RD, MM, AS, JG, and SM wrote the manuscript. RD will act as guarantor. All authors have discussed the results, commented on the manuscript, and agreed to be accountable for all aspects of the work in ensuring that questions related to the accuracy or integrity of any part of the work are appropriately investigated and resolved.

## Conflict of Interest

The authors declare that the research was conducted in the absence of any commercial or financial relationships that could be construed as a potential conflict of interest.

## References

[1] Overweight and obesity. WHO Fact Sheets. WHO. Available online at: https://www.who.int/news-room/fact-sheets/detail/obesity-and-overweight (accessed on July 19, 2019).

[2] Ranjani H Mehreen TS Pradeepa R Anjana RM Garg R Anand K Mohan V. Epidemiology of childhood overweight & obesity in India: a systematic review. Indian J Med Res. (2016) 143:160–74. 10.4103/0971-5916.180203 27121514 PMC4859125

[3] Das RR Mangaraj M Panigrahi SK Satapathy AK Mahapatro S Ray PS. Metabolic syndrome and insulin resistance in schoolchildren from a developing country. Front Nutr. (2020) 7:31. 10.3389/fnut.2020.00031 32296710 PMC7141174

[4] Tharkar S Devarajan A Kumpatla S Muthukumaran P Viswanathan V. Insulin resistance and cardio metabolic abnormalities among overweight South Indian children: chennai slim and fit programme. Indian J Community Med. (2013) 38:121–2. 10.4103/0970-0218.112452 23878428 PMC3714941

[5] Misra A Khurana L. The metabolic syndrome in South Asians: epidemiology, determinants, and prevention. Metab Syndr Relat Disord. (2009) 7:497–514. 10.1089/met.2009.0024 19900153

[6] Misra A Vikram NK Arya S Pandey RM Dhingra V Chatterjee A. High prevalence of insulin resistance in postpubertal Asian Indian children is associated with adverse truncal body fat patterning, abdominal adiposity and excess body fat. Int J Obes Relat Metab Disord. (2004) 28:1217–26. 10.1038/sj.ijo.0802704 15314636

[7] Tagi VM Giannini C Chiarelli F. Insulin resistance in children. Front Endocrinol. (2019) 10:342. 10.3389/fendo.2019.00342 PMC6558106 31214120

[8] Gepstein V Weiss R. Obesity as the main risk factor for metabolic syndrome in children. Front Endocrinol. (2019) 10:568. 10.3389/fendo.2019.00568 PMC6706788 31474943

[9] Lentferink YE Elst MAJ Knibbe CAJ van der Vorst MMJ. Predictors of insulin resistance in children versus adolescents with obesity. J Obes. (2017) 2017:3793868. 10.1155/2017/3793868 29375912 PMC5742469

[10] Gupta A Sachdeva A Mahajan N Gupta A Sareen N Pandey RM. Prevalence of pediatric metabolic syndrome and associated risk factors among school-age children of 10–16 years living in district Shimla, Himachal Pradesh, India. Indian J Endocrinol Metab. (2018) 22:373–8. 10.4103/ijem.IJEM_251_17 30090730 PMC6063189

[11] Levy-Marchal C Arslanian S Cutfield W Sinaiko A Druet C Marcovecchio ML. Insulin resistance in children: consensus, perspective, and future directions. J Clin Endocrinol Metab. (2010) 95:5189–98. 10.1210/jc.2010-1047 20829185 PMC3206517

[12] Khadilkar A Ekbote V Chiplonkar S Khadilkar V Kajale N Kulkarni S. Waist circumference percentiles in 2–18 year old Indian children. J Pediatr. (2014) 164:1358–62. 10.1016/j.jpeds.2014.02.018 24655536

[13] Flynn JT Kaelber DC Baker-Smith CM Blowey D Carroll AE Daniels SR. Clinical practice guideline for screening and management of high blood pressure in children and adolescents. Pediatrics. (2017) 140:e20171904. 10.1542/peds.2017-1904 28827377

[14] Indian Academy of Pediatrics Growth Charts Committee Khadilkar V Yadav S Agrawal KK Tamboli S Banerjee M. Revised IAP growth charts for height, weight and body mass index for 5- to 18-year-old Indian children. Indian Pediatr. (2015) 52:47–55. 10.1007/s13312-015-0566-5 25638185

[15] Vikram NK Misra A Pandey RM Dwivedi M Luthra K. Adiponectin, insulin resistance, and C-reactive protein in postpubertal Asian Indian adolescents. Metabolism. (2004) 53:1336–41. 10.1016/j.metabol.2004.05.010 15375791

[16] Khalil A Gupta S Madan A Venkatesan M. Lipid profile norms in Indian children. Indian Pediatr. (1995) 32:1177–80. 8772866

[17] Zimmet P Alberti KG Kaufman F Tajima N Silink M Arslanian S. The metabolic syndrome in children and adolescents - an IDF consensus report. Pediatr Diabetes. (2007) 8:299–306. 10.1111/j.1399-5448.2007.00271.x 17850473

[18] Steinberger J Daniels SR Eckel RH Hayman L Lustig RH McCrindle B. Progress and challenges in metabolic syndrome in children and adolescents: a scientific statement from the American Heart Association Atherosclerosis, Hypertension, and Obesity in the Young Committee of the Council on Cardiovascular Disease in the Young; Council on Cardiovascular Nursing; and Council on Nutrition, Physical Activity, and Metabolism. Circulation. (2009) 119:628–47. 10.1161/CIRCULATIONAHA.108.191394 19139390

[19] Reisinger C Nkeh-Chungag BN Fredriksen PM Goswami N. The prevalence of pediatric metabolic syndrome-a critical look on the discrepancies between definitions and its clinical importance. Int J Obes. (2021) 45:12–24. 10.1038/s41366-020-00713-1 33208861 PMC7752760

[20] Matthews DR Hosker JP Rudenski AS Naylor BA Treacher DF Turner RC. Homeostasis model assessment: insulin resistance and beta-cell function from fasting plasma glucose and insulin concentrations in man. Diabetologia. (1985) 28: 412–9. 10.1007/BF00280883 3899825

[21] Singh Y Garg MK Tandon N Marwaha RK. A study of insulin resistance by HOMA-IR and its cut-off value to identify metabolic syndrome in urban Indian adolescents. J Clin Res Pediatr Endocrinol. (2013) 5:245–51. 10.4274/Jcrpe.1127 24379034 PMC3890224

[22] Yin J Li M Xu L Wang Y Cheng H Zhao X Mi J. Insulin resistance determined by Homeostasis Model Assessment (HOMA) and associations with metabolic syndrome among Chinese children and teenagers. Diabetol Metab Syndr. (2013) 5:71. 10.1186/1758-5996-5-71 24228769 PMC3833654

[23] Rodea-Montero ER Evia-Viscarra ML Apolinar-Jiménez E. Waist-to-height ratio is a better anthropometric index than waist circumference and BMI in predicting metabolic syndrome among obese Mexican adolescents. Int J Endocrinol. (2014) 2014:195407. 10.1155/2014/195407 25574166 PMC4276350

[24] Patnaik L Pattanaik S Sahu T Rao EV. Overweight and obesity among adolescents, a comparative study between government and private schools. Indian Pediatr. (2015) 52:779–81. 10.1007/s13312-015-0716-9 26519713

[25] Friend A Craig L Turner S. The prevalence of metabolic syndrome in children: a systematic review of the literature. Metab Syndr Relat Disord. (2013) 11:71–80. 10.1089/met.2012.0122 23249214

[26] Atabek ME Pirgon O. Assessment of insulin sensitivity from measurements in fasting state and during an oral glucose tolerance test in obese children. J Pediatr Endocrinol Metab. (2007) 20:187–95. 10.1515/JPEM.2007.20.2.187 17396435

[27] Keskin M Kurtoglu S Kendirci M Atabek ME Yazici C. Homeostasis model assessment is more reliable than the fasting glucose/insulin ratio and quantitative insulin sensitivity check index for assessing insulin resistance among obese children and adolescents. Pediatrics. (2005) 115:e500–3. 10.1542/peds.2004-1921 15741351

[28] Wickramasinghe VP Arambepola C Bandara P Abeysekera M Kuruppu S Dilshan P Dissanayake BS. Insulin resistance in a cohort of 5–15 year old children in urban Sri Lanka. BMC Res Notes. (2017) 10:347. 10.1186/s13104-017-2658-x 28754153 PMC5534057

[29] Denney-Wilson E Cowell CT Okely AD Hardy LL Aitken R Dobbins T. Associations between insulin and glucose concentrations and anthropometric measures of fat mass in Australian adolescents. BMC Pediatr. (2010) 10:58. 10.1186/1471-2431-10-58 20701807 PMC2927904

[30] van der Aa MP Fazeli Farsani S Knibbe CA de Boer A van der Vorst MM. Population-based studies on the epidemiology of insulin resistance in children. J Diabetes Res. (2015) 2015:362375. 10.1155/2015/362375 26273668 PMC4530262

[31] Manios Y Moschonis G Kourlaba G Bouloubasi Z Grammatikaki E Spyridaki A. Prevalence and independent predictors of insulin resistance in children from Crete, Greece: the children study. Diabet Med. (2008) 25:65–72. 10.1111/j.1464-5491.2007.02318.x 18028438

[32] Grant AM Taungapeau FK McAuley KA Taylor RW Williams SM Waldron MA. Body mass index status is effective in identifying metabolic syndrome components and insulin resistance in Pacific Island teenagers living in New Zealand. Metabolism. (2008) 57:511–6. 10.1016/j.metabol.2007.11.013 18328353

[33] Romualdo MC Nóbrega FJ Escrivão MA. Insulin resistance in obese children and adolescents. J Pediatr. (2014) 90:600–7. 10.1016/j.jped.2014.03.005 25019650

[34] McAuley KA Williams SM Mann JI Walker RJ Lewis-Barned NJ Temple LA. Diagnosing insulin resistance in the general population. Diabetes Care. (2001) 24:460–4. 10.2337/diacare.24.3.460 11289468

[35] Viner RM Segal TY Lichtarowicz-Krynska E Hindmarsh P. Prevalence of the insulin resistance syndrome in obesity. Arch Dis Child. (2005) 90:10–4. 10.1136/adc.2003.036467 15613503 PMC1720077

[36] Styne DM Arslanian SA Connor EL Farooqi IS Murad MH Silverstein JH. Pediatric obesity-assessment, treatment, and prevention: an endocrine society clinical practice guideline. J Clin Endocrinol Metab. (2017) 102:709–57. 10.1210/jc.2016-2573 28359099 PMC6283429

